# Impact of Time to Castration Resistance on Survival in Metastatic Hormone Sensitive Prostate Cancer Patients in the Era of Combination Therapies

**DOI:** 10.3389/fonc.2021.659135

**Published:** 2021-04-23

**Authors:** Mike Wenzel, Felix Preisser, Benedikt Hoeh, Maria Schroeder, Christoph Würnschimmel, Thomas Steuber, Hans Heinzer, Severine Banek, Marit Ahrens, Andreas Becker, Pierre I. Karakiewicz, Felix K. H. Chun, Luis A. Kluth, Philipp Mandel

**Affiliations:** ^1^ Department of Urology, University Hospital Frankfurt, Goethe University Frankfurt am Main, Frankfurt, Germany; ^2^ Cancer Prognostics and Health Outcomes Unit, Division of Urology, University of Montreal Health Center, Montreal, QC, Canada; ^3^ Martini-Klinik Prostate Cancer Center, University Hospital Hamburg-Eppendorf, Hamburg, Germany; ^4^ Department of Hematology and Oncology, University Hospital Frankfurt, Frankfurt, Germany

**Keywords:** mortality, survival, castration resistance, metastatic prostate cancer, CRPC

## Abstract

**Background:**

To evaluate the impact of time to castration resistance (TTCR) in metastatic hormone-sensitive prostate cancer (mHSPC) patients on overall survival (OS) in the era of combination therapies for mHSPC.

**Material and Methods:**

Of 213 mHSPC patients diagnosed between 01/2013-12/2020 who subsequently developed metastatic castration resistant prostate cancer (mCRPC), 204 eligible patients were analyzed after having applied exclusion criteria. mHSPC patients were classified into TTCR <12, 12-18, 18-24, and >24 months and analyzed regarding OS. Moreover, further OS analyses were performed after having developed mCRPC status according to TTCR. Logistic regression models predicted the value of TTCR on OS.

**Results:**

Median follow-up was 34 months. Among 204 mHSPC patients, 41.2% harbored TTCR <12 months, 18.1% for 12-18 months, 15.2% for 18-24 months, and 25.5% for >24 months. Median age was 67 years and median PSA at prostate cancer diagnosis was 61 ng/ml. No differences in patient characteristics were observed (all p>0.05). According to OS, TTCR <12 months patients had the worst OS, followed by TTCR 12-18 months, 18-24 months, and >24 months, in that order (p<0.001). After multivariable adjustment, a 4.07-, 3.31-, and 6.40-fold higher mortality was observed for TTCR 18-24 months, 12-18 months, and <12 months patients, relative to TTCR >24 months (all p<0.05). Conversely, OS after development of mCRPC was not influenced by TTCR stratification (all p>0.05).

**Conclusion:**

Patients with TTCR <12 months are at the highest OS disadvantage in mHSPC. This OS disadvantage persisted even after multivariable adjustment. Interestingly, TTCR stratified analyses did not influence OS in mCRPC patients.

## Introduction

Prostate cancer is the most common cancer in men and is moreover the second and third most common cause of cancer-specific mortality in the United States and Europe (1–3). Even though survival rates are excellent in localized prostate cancer, metastatic prostate cancer is a palliative situation (4, 5). For several decades, androgen deprivation therapy (ADT) has been the agent of choice in the treatment of metastatic hormone-sensitive prostate cancer (mHSPC) (6). With the publication of the GETUG-AFU 15 trial in 2013, in additional to ADT, several combination therapies, such as abiraterone, docetaxel, apalutamide, or enzalutamide, were approved for treatment in mHSPC; these combination therapies showed benefit according to overall survival (OS) and progression-free survival (PFS), especially in high-volume mHSPC (7–13). In consequence, one aim in the treatment of mHSPC is to delay progression to metastatic castration resistant prostate cancer (mCRPC) (14).

Recently, two Japanese studies focused on the effect of differences in time to castration resistance (TTCR) on OS (15, 16). However, these studies relied exclusively on patients treated with either ADT alone or combination of ADT plus bicalutamide in mHSPC. In consequence, little if anything is known about the impact of differences in TTCR on survival in the era of the above-described combination therapies, especially in European mHSPC patients.

We addressed this void and relied on our institutional metastatic prostate cancer database since the year of the first publication of combination therapy in mHSPC, to investigate the effect of TTCR on OS. We hypothesized that differences in TTCR will result in differences in OS rates but may not influence OS after development of mCRPC even in the era of combination therapies.

## Materials and Methods

### Study Population

After approval of the local ethics committee, all patients with mHSPC and subsequent mCRPC who were diagnosed since 2013 (year of the first publication of combination therapy in mHSPC) at the Department of Urology, University Hospital Frankfurt, Germany, were retrospectively identified (n=213) (12). All patients who were diagnosed between 01/2013 and 12/2020 were included in the current study. Exclusion criteria were unknown follow-up status or unknown status regarding the time point of progression to mCRPC (n=9). These selection criteria resulted in 204 eligible mHSPC patients.

### mCRPC Definition

mCRPC status was defined in accordance with the EAU guidelines (4): PSA progression of three consecutive rises of PSA values or a 50% increase of absolute PSA values over the PSA nadir under mHSPC treatment combined with a testosterone level <50 ng/dl. Additionally, a radiographic progression with at least two new bone metastases or one visceral metastasis was defined as mCRPC (17). For TTCR analyses, the duration from the beginning of the treatment in mHSPC to the first stated date of mCRPC status was calculated. For OS analyses, the duration from the date beginning the treatment in mHSPC or mCRPC to patients’ death of any course was computed.

### Statistical Analysis

Descriptive statistics included frequencies and proportions for categorical used variables. Medians and interquartile ranges (IQR) were reported for all used continuously variables. The Chi-square test was used to test for statistical significance in proportions’ differences. The t-test and Kruskal-Wallis test examined the statistical significance of distributions’ differences.

mHSPC patients were stratified into TTCR <12, 12-18, 18-24, and >24 months and accordingly analyzed with regard to OS. In the second set of the analyses, OS analyses were performed in the four TTCR subgroups since development of mCRPC status. Univariable and multivariable Cox regression models were fitted to predict the value of TTCR on OS in both analyses. All variables with p<0.25 in univariable analyses were considered for multivariable analyses, as recently recommended (18).

All tests were two-sided with a level of significance set at p<0.05. R software environment for statistical computing and graphics (version 3.4.3) was used for all analyses (19).

## Results

### Descriptive Baseline Characteristics

Median follow-up duration was 34 months. Among 204 eligible mHSPC patients, 41.2% (n=84) had a TTCR of <12 months, 18.1% (n=37) of 12-18 months, 15.2% (n=31) of 18-24 months, and 25.5% (n=152) of >24 months (**Table 1**). Median age and PSA at prostate cancer diagnosis did not differ between the four TTCR groups. Overall, median age was 67 years (IQR 61-73) and median PSA was 61 ng/ml (IQR 15-294, both p≥0.2). Moreover, the Eastern Cooperative Oncology Group (ECOG) distribution did not differ between the TTCR subgroups (p=0.2). Similarly, proportions of primary metastatic patients, high-volume metastatic burden according to CHAARTED criteria, and number and proportions of metastatic sides did not differ between the four TTCR subgroups (all p≥0.2). Patients with TTCR <12 months less frequently received local therapy for primary tumor with radical prostatectomy or radiation therapy than TTCR 12-18, 18-24, and >24 months, without reaching statistical significance (20.2, 27.0, 38.7, and 26.9%, p=0.3). Moreover, differences in combination therapies for mHSPC existed (p=0.01). Overall, in mHSPC patients, 55.4% (n=113) of patients received treatment with ADT vs. 22.5% (n=46) docetaxel vs. 14.2% (n=29) abiraterone vs. 3.4% (n=7) enzalutamide vs. 4.4% (n=9) other treatments. Median treatment therapy lines in mCRPC were 2 (IQR 1-3). Detailed characteristics of all four TTCR subgroups are summarized in **Table 1**.

**Table 1:** Descriptive characteristics of 204 metastatic hormone-sensitive prostate cancer (mHSPC) patients, diagnosed between 2013-2020 at the University Hospital Frankfurt, stratified according to time to castration resistance.

Variable		Overall n=204	<12 months n=84 (41.2%)	12-18 months n=37 (18.1%)	18-24 months n=31 (15.2%)	>24 months n=52 (25.5%)	P value
Age at prostate cancer diagnosis	Median (IQR)	67 (61-73)	67 (62-73)	66 (61-71)	65 (57-72)	69 (62-75)	0.2
PSA at prostate cancer diagnosis	Median (IQR)	61 (15-294)	61 (14-194)	166 (37-397)	55 (17-218)	44 (14-353)	0.8
PSA at mCRPC	Median (IQR)	20 (5-70)	13 (4-63)	32 (9-94)	39 (13-94)	19 (3-38)	0.6
Primary metastatic	Yes	98 (48.0)	44 (52.4)	15 (40.5)	11 (35.5)	28 (53.8)	0.2
	No	104 (51.0)	40 (47.6)	21 (56.8)	20 (64.5)	23 (44.2)	
If primary metastatic	M1a	21 (10.3)	7 (8.3)	5 (13.5)	4 (12.9)	5 (9.6)	0.3
	M1b/c	76 (37.3)	36 (42.9)	10 (27.0)	7 (22.6)	23 (44.2)	
Tumor burden according to CHAARTED	Low Burden	36 (17.6)	19 (22.6)	4 (10.8)	3 (9.7)	10 (19.2)	0.9
	High Burden	41 (20.1)	18 (21.4)	6 (16.2)	4 (12.9)	13 (25.0)	
Visceral metastases	No	141 (69.1)	64 (76.2)	23 (62.2)	21 (67.7)	33 (63.5)	0.2
	Yes	12 (5.9)	4 (4.8)	1 (2.7)	1 (3.2)	6 (11.5)	
If M1a at diagnosis: Number of metastases	Median (IQR)	2 (1-3)	1 (1-2)	2 (1-3)	4 (2-6)	2 (1-3)	0.07
If M1b at diagnosis: Number of metastases	Median (IQR)	3 (1-7)	3 (1-6)	4 (2-7)	3 (1-5)	4 (3-8)	0.7
If M1c at diagnosis: Number of metastases	Median (IQR)	2 (1-2)	2 (2-2)	1 (1-1)	1 (1-1)	2 (1-2)	0.1
ECOG at prostate cancer diagnosis	0	88 (43.1)	34 (40.5)	14 (37.8)	14 (45.2)	26 (50.0)	0.2
	1	71 (34.8)	29 (34.5)	16 (43.2)	13 (41.9)	13 (25)	
	≥2	13 (6.4)	9 (10.7)	2 (5.4)	1 (3.2)	1 (1.9)	
Gleason Score at diagnosis	6-7	34 (16.7)	12 (14.3)	3 (8.1)	9 (29.0)	10 (19.2)	0.1
	8-10	151 (74.0)	64 (76.2)	30 (81.1)	19 (61.3)	38 (73.1)	
Local therapy	None/Other	151 (74.0)	67 (79.8)	27 (73.0)	19 (61.3)	38 (73.1)	0.3
	Yes	53 (26.0)	17 (20.2)	10 (27.0)	12 (38.7)	14 (26.9)	
Therapy for mHSPC	ADT alone	113 (55.4)	46 (54.8)	13 (35.1)	22 (71.0)	32 (61.5)	<0.01
	Abiraterone	29 (14.2)	10 (11.9)	5 (13.5)	4 (12.9)	10 (19.2)	
	Docetaxel	46 (22.5)	21 (25.0)	18 (48.6)	4 (12.9)	3 (5.8)	
	Enzalutamide	7 (3.4)	3 (3.6)	1 (2.7)	1 (3.2)	2 (3.8)	
	Other	9 (4.4)	4 (4.8)	0 (0)	0 (0)	5 (9.6)	
Therapy for mCRPC	Abiraterone	68 (33.3)	28 (33.3)	11 (29.7)	12 (38.7)	17 (32.7)	0.8
	Docetaxel	27 (13.2)	13 (15.5)	5 (13.5)	1 (3.2)	8 (15.4)	
	Enzalutamide	35 (17.2)	11 (13.1)	7 (18.9)	8 (25.8)	9 (17.3)	
	ADT/Other/None	74 (36.3)	32 (38.1)	14 (37.8)	10 (32.3)	18 (34.6)	
Therapy lines in mCRPC	Median (IQR)	2 (1-3)	1 (1-3)	2 (1-3)	2 (1-3)	1 (1-3)	0.2

IQR, Interquartile range; PSA, Prostate-specific antigen; mCRPC, metastatic castration resistance prostate cancer; ECOG, Eastern Cooperative Oncology Group; ADT, Androgen deprivation therapy.

### Survival in mHSPC Patients According to TTCR

**Figure 1:**
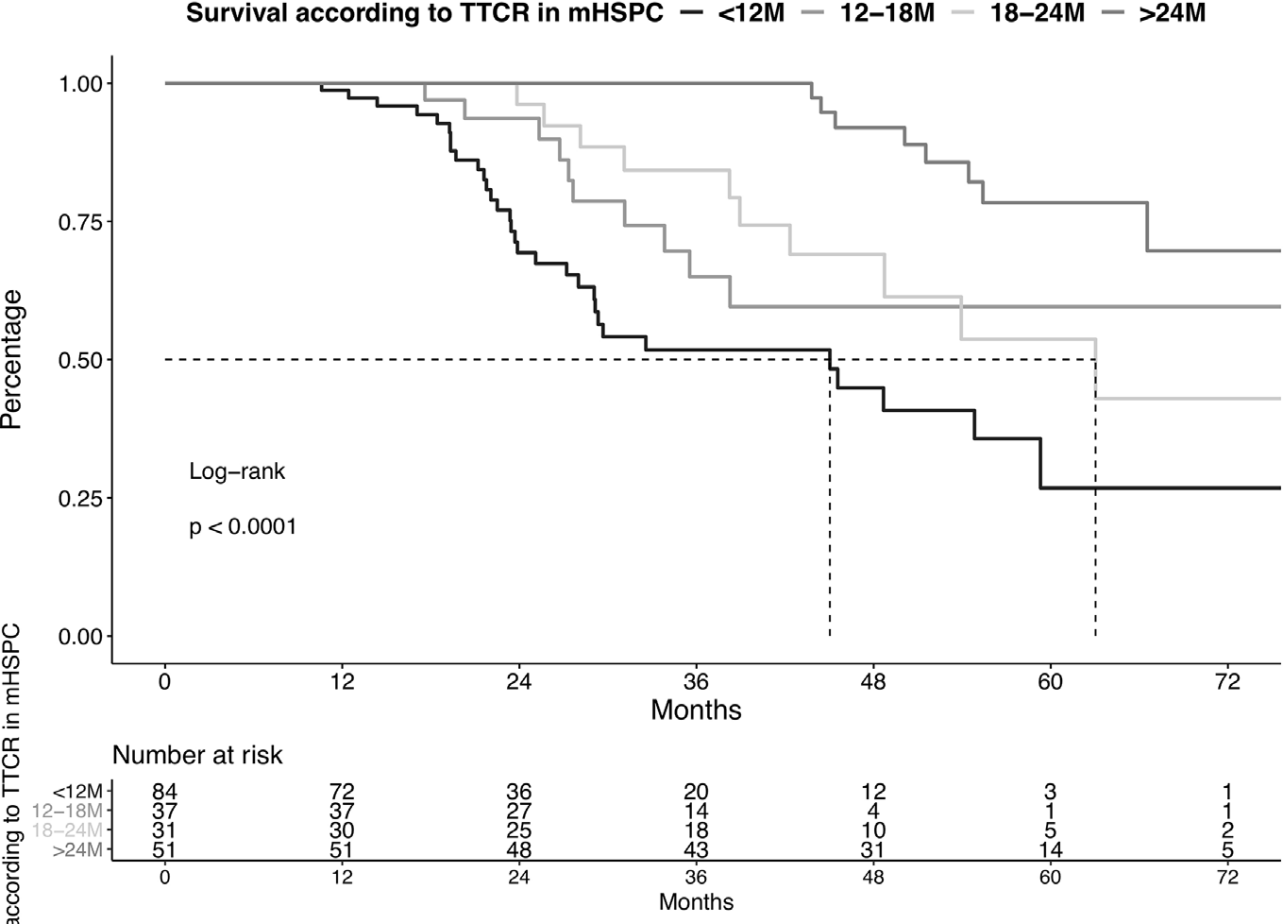
Kaplan-Meier plot illustrating overall survival in metastatic hormone-sensitive prostate cancer (mHSPC) patients diagnosed between 2013-2020, stratified according to time to castration resistance (TTCR). The follow-up starts from the beginning of the therapy in mHSPC. M, Months.

Patients with TTCR <12 months had the worst OS, followed by TTCR 12-18 months, 18-24 months, and >24 months, in that order (**Figure 1**, p<0.001). These OS differences translated into a 2.93-fold (confidence interval [CI]: 1.15-7.34, p=0.02), 3.78-fold (CI: 1.47-9.72, p<0.01), and 6.95-fold (CI 3.15-15.32, p<0.001) higher risk of overall mortality for TTCR 18-24 months, 12-18 months, and <12 months patients, relative to TTCR >24 months patients (**Table 2**).

**Table 2:** Univariable and multivariable Cox regression models predicting overall survival in metastatic hormone-sensitive prostate cancer (mHSPC) patients according to time to castration resistance (TTCR).

	Univariable			Multivariable		
	HR	95% CI	P-value	HR	95% CI	P-value
TTCR >24 months (Ref.)	1	–	–	1	–	–
TTCR 18-24 months	2.93	1.15-7.43	0.02	3.92	1.42-10.84	<0.01
TTCR 12-18 months	3.78	1.47-9.72	<0.01	3.59	1.28-10.08	0.01
TTCR <12 months	6.95	3.15-15.32	<0.001	7.14	2.85-17.88	<0.001
PSA at diagnosis	1.00	1.00-1.00	1			
No local therapy (Ref.)	1	–	–			
Local therapy	0.81	0.44-1.50	0.5			
Number of bone metastases in mHSPC	1.01	0.91-1.11	0.9			
Number of visceral metastases in mHSPC	1.12	0.59-2.10	0.7			
Age at diagnosis	1.00	0.97-1.03	1			
ECOG 0 (Ref.)	1	–	–	1	–	–
ECOG 1	1.78	1.01-3.14	0.047	1.67	0.86-4.45	0.1
ECOG ≥2	5.55	2.48-12.39	<0.001	4.77	2.05-11.12	<0.001
Gleason score 6-7 (Ref.)	1	–	–	1	–	–
Gleason score 8-10	2.59	1.03-6.52	0.043	2.03	0.75-5.52	0.2
ADT (Ref.)	1	–	–	1	–	–
Abiraterone/Enzalutamide	1.24	0.61-2.51	0.6	1.95	0.86-4.45	0.4
Docetaxel	1.72	0.89-3.34	0.1	1.37	0.67-2.81	0.4
Secondary metastatic (Ref.)	1	–	–			
Primary metastatic	1.20	0.72-2.00	0.5			
Therapy lines in mCRPC	0.88	0.72-1.09	0.24	0.83	0.66-1.06	0.1

Due to sample size limitations, abiraterone and enzalutamide were grouped. PSA, Prostate-specific antigen; ECOG, Eastern Cooperative Oncology Group; mCRPC, metastatic castration resistance prostate cancer; HR, Hazard ratio; CI, Confidence interval.

After multivariable adjustment for patient and prostate cancer characteristics, the significant OS disadvantages persisted in all three TTCR subgroups. Specifically, hazard ratios (HR) of 3.92 (CI 1.42-10.84, p<0.01), 3.59 (CI 1.28-10.08, p=0.01), and 7.14 (CI 2.85-17.88, p<0.001) were recorded for TTCR 18-24 months, 12-18 months, and <12 months patients, relative to patients with TTCR >24 months patients. Moreover, ECOG ≥2 was an independent predictor for worse OS (HR: 4.45, p<0.001).

### Survival in mCRPC Patients According to TTCR

**Figure 2:**
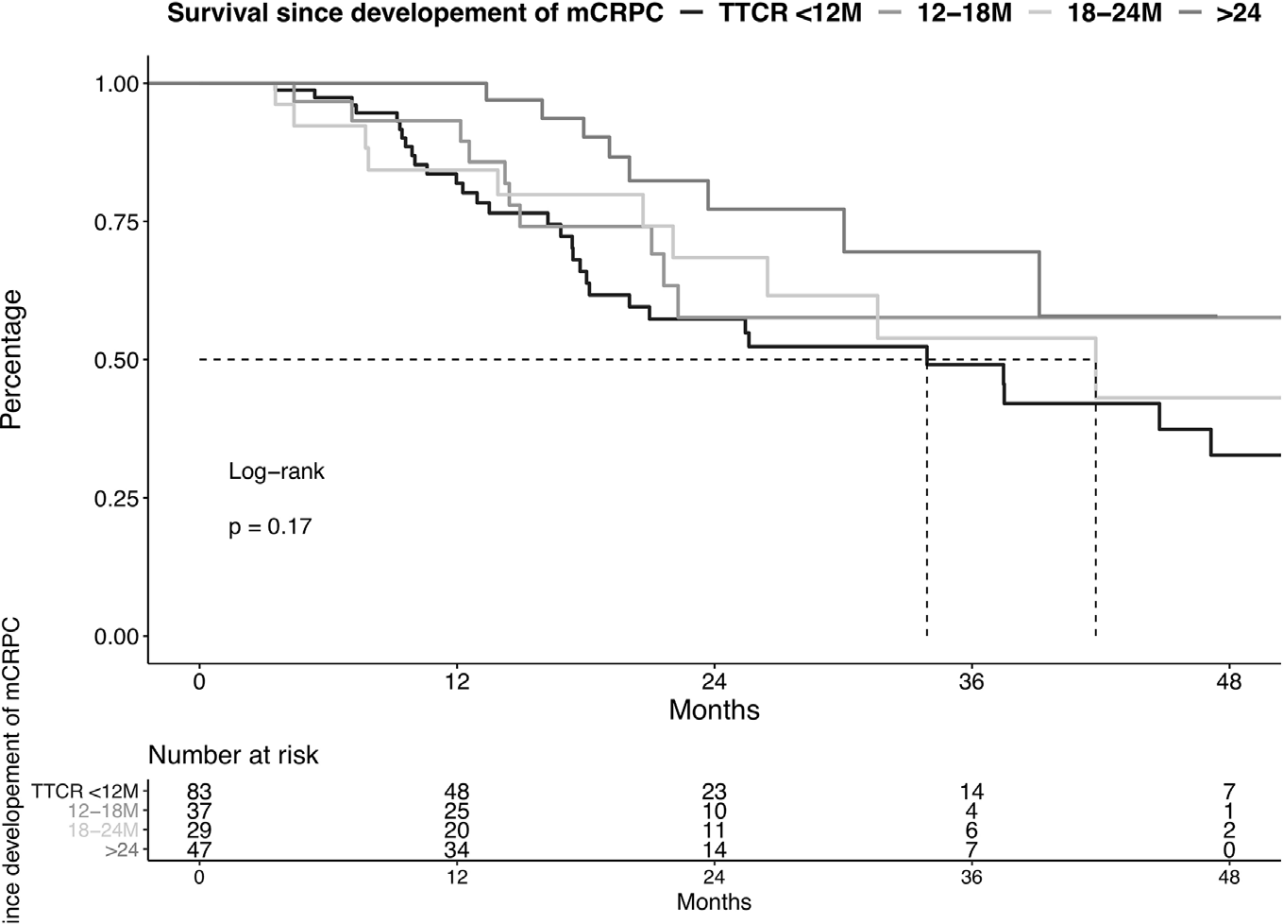
Kaplan-Meier plot illustrating overall survival in metastatic castration resistance prostate cancer (mCRPC) patients diagnosed between 2013-2020, stratified according to the time to castration resistance (TTCR). M, Months.

OS did not differ in all four examined TTCR groups after development of mCRPC (**Figure 2**, p=0.2). Moreover, after multivariable adjustment for patient and prostate cancer characteristics, no statistically significant OS differences were observed between the TTCR subgroups 18-24 months, 12-18 months, and <12 months, relative to TTCR >24 months patients (all p>0.05, **Table 3**). Conversely, ECOG 1 and ≥2, as well as the number of visceral metastases, were independently associated with worse OS (HRs: 2.95, 9.52, 2.82, all p ≤ 0.02).

**Table 3:** Univariable and multivariable Cox regression models predicting overall survival in metastatic castration resistant prostate cancer patients according to time to castration resistance (TTCR).

	Univariable			Multivariable		
	HR	95% CI	P-value	HR	95% CI	P-value
TTCR >24 months (Ref.)	1	–	–	1	–	–
TTCR 18-24 months	1.84	0.73-4.68	0.2	4.98	0.70-35.35	0.1
TTCR 12-18 months	1.78	0.70-4.52	0.2	2.01	0.36-11.33	0.4
TTCR <12 months	2.38	0.98-5.19	0.059	4.00	0.91-17.78	0.07
PSA at diagnosis	1.00	1.00-1.00	0.9			
No local therapy (Ref.)	1	–	–			
Local therapy	0.94	0.51-1.75	0.9			
Number of bone metastases in mHSPC	1.03	0.93-1.14	0.6			
Number of visceral metastases in mHSPC	1.63	0.89-2.98	0.1	2.82	1.34-5.93	<0.01
Age at diagnosis	1.01	0.98-1.04	0.5			
ECOG 0 (Ref.)	1	–	–	1	–	–
ECOG 1	1.65	0.93-2.93	0.08	2.95	1.18-7.40	0.02
ECOG ≥2	4.32	1.94-9.63	<0.01	9.52	3.24-27.99	<0.01
Gleason score 6-7 (Ref.)	1	–	–	1	–	–
Gleason score 8-10	2.15	0.86-5.40	0.1	1.03	0.29-3.67	1
ADT (Ref.)	1					
Abiraterone/Enzalutamide	1.44	0.69-2.53	0.3			
Docetaxel	1.32	0.69-2.53	0.4			
Secondary metastatic (Ref.)	1					
Primary metastatic	1.42	0.85-2.37	0.2	1.78	0.76-4.18	0.2
Therapy lines in mCRPC	0.78	0.63-0.97	0.03	0.71	0.50-1.01	0.055

Due to sample size limitations, abiraterone and enzalutamide were grouped. PSA, Prostate-specific antigen; mHSPC, Metastatic hormone-sensitive prostate cancer; ECOG, Eastern Cooperative Oncology Group; mCRPC, Metastatic castration resistant prostate cancer; HR, Hazard ratio; CI, Confidence interval.

## Discussion

We hypothesized that, even in the era of combination therapies for mHSPC, differences in TTCR may result in differences in OS rates. We tested this hypothesis in our institutional metastatic prostate cancer database and focused exclusively on patients diagnosed since the year of the first publication of combination therapy (2013) in mHSPC and made several noteworthy observations.

First, we observed important differences regarding OS in the four examined TTCR groups. Specifically, TTCR <12 months patients exhibited the worst OS, while TTCR >24 patients exhibited the best OS in mHSPC. Remarkably, patients with TTCR <12 months had a 6.95-fold higher risk of overall mortality relative to patients with TTCR >24 months. It is particularly important to emphasize that this observation was also made after controlling and adjusting for patient and tumor characteristics in multivariable analyses, where all three TTCR groups were at an OS disadvantage relative to TTCR >24 months patients. These observations are in line with the previous publication by Miyake et al. Specifically, Miyake et al. also observed a higher OS in patients with shorter TTCR (15). Moreover, they also found that patients with the longest TTCR had the best OS. However, this study relied on a TTCR stratification of <6 vs. 6-12 vs. 12-18 vs. >18 months. Thus, direct comparisons of OS rates cannot be made. Due to the patients’ TTCR distribution in our cohort, our stratification relied on <12, 12-18, 18-24, and >24 months. This stratification is particularly important, considering that in the study by Miyake et al., all patients received ADT only, while in our cohort approximately 45% received combination therapy in mHSPC. Therefore, compared with the study by Miyake et al., we suspected a longer TTCR in the current study, such as proven in the CHAARTED trial, where combination therapy was used (TTCR for docetaxel and ADT vs. ADT alone: 19.4 vs. 11.7 months). Nonetheless, the clinical implications might be the same in both studies. Moreover, a recent report of 283 mHSPC Japanese patients also observed that TTCR <12 months is associated with worse survival (16). Similarly, Bournakis et al. also investigated an OS disadvantage in patients with TTCR ≤24 months in a more historical cohort (1996-2009) (20). In summary, our observations are particularly important since they validate these previous findings in a large contemporary European metastatic patient cohort of which a large proportion of patients received combination therapy in mHSPC.

Second, we also made important observations according to OS analyses in mCRPC patients stratified according to TTCR. Specifically, we observed no differences in OS between the four examined TTCR subgroups when OS rates were compared after development of mCRPC. It is even more important to emphasize that these observations persisted even after adjustment for patient and tumor characteristics in multivariable analyses. This observation is also in agreement with the publication of Miyake et al., which focused exclusively on ADT patients (15). Specifically, in the current study, no OS differences were observed between all TTCR subgroups since the development of mCRPC. This observation may lead to the assumption that prolonging OS is most effective when TTCR is prolonged in first-line therapy of mHSPC patients. Therefore, clinicians should be aware of the fact that a prolonged TTCR may be a surrogate for OS.

It is also of note that in both mHSPC and mCRPC OS analyses, ECOG ≥2 was independently associated with worse OS after adjusting for patient and tumor characteristics in logistic regression models. These observations are not surprising but validate our survival analyses with regard to patients’ frailty. Moreover, these observations are important since the adjustment for ECOG status equalized differences in the ECOG status distribution between all four examined TTCR subgroups in both OS analyses. Therefore, our multivariable OS results can be interpreted regardless of patients’ frailty that may have led to other cause mortality.

Third, previous publications reported differences in baseline characteristics of patients with different TTCR. For example, Miyake et al. investigated that patients with shorter TTCR had higher proportions of visceral metastasis (15). Additionally, several studies aimed to investigate risk factors for progression to mCRPC in mHSPC patients. Several risk factors such as PSA, Gleason score, or time to PSA nadir in mHSPC treatment were found to be such predictors (21–26). Despite non-significant differences between the four examined TTCR subgroups, possibly due to sample size limitations, we also found interesting trends. For example, as observed in the current study cohort, higher proportions of Gleason score 8-10 were observed in the subgroups TTCR <12 and 12-18 compared to TTCR 18-24 and >24. Moreover, we also found that patients with TTCR <12 months had the worst distribution of tumor burden of visceral metastases, when visceral metastases were found at mHSPC diagnosis. Moreover, number of visceral metastases were a significant predictor for worse OS in mCRPC. Additionally, patients with TTCR <12 months less frequently received local therapies with radical prostatectomy or radiation therapy for the primary tumor, relative to patients with TTCR 12-18, 18-24, or >24 months. Although there was no statistical significance of these variables, these findings may indicate one of the reasons why patients exhibited the shortest TTCR. However, OS differences between TTCR groups cannot be explained by differences in patient or baseline tumor characteristics alone. It is very likely that other factors, such as genetic differences or gene mutations, play a crucial role in TTCR for which we could unfortunately not account for (27, 28).

Our study has several limitations and needs to be considered in the light of its retrospective, single-center design. Although only 4.2% (n=9) of all identified mHSPC patients were excluded after applying inclusion criteria of the current study, a selection bias cannot be completely ruled out. Second, differences between some variables might result in a lack of significance due to limitations in sample size or missing baseline information (e.g., staging modalities) for some patients. Moreover, no treatment-specific TTCR analyses in mHSPC patients could be performed due to limitations in sample size and differences in baseline prostate cancer characteristics between the treatment groups. As the first study analyzing combination therapy in mHSPC – the GETUF-AFU15 study - was published in 2013, the current study focused on the most contemporary patients treated with combination therapy in mHSPC between 2013 to 2020 (12). In consequence, not all patients had directly received new combination therapies upfront since 2013. This circumstance might have led to a heterogenous patient cohort, where approximately half of the included patients received combination therapy in mHSPC, which still reflects current daily practice in many European countries. Moreover, differences in patients’ therapies in mCRPC may have affected OS and might be linked to prior therapies in mHSPC. However, since we aimed to investigate the effect of TTCR on OS regardless of the administered treatment, different treatments may have influenced TTCR, but did not bias the results and the implications of this work. However, to adjust for possible OS differences related to treatments in mHSPC, we included combination therapies as a covariate in all Cox regression models. Finally, since no previous study relied on patients who received combination therapy for mHSPC, the chosen TTCR cut-offs need to be further validated in patient cohorts that were treated with combination therapy.

Taken together, our study demonstrates that TTCR also affects OS in mHSPC patients in the era of combination therapies for mHSPC. More specifically, patients with TTCR <12 months are at highest risk of overall mortality, while TTCR >24 months patients exhibited the longest OS. Moreover, these findings were also observed after controlling for patient and prostate cancer characteristics in multivariable adjustment. Finally, stratification according to TTCR in mCRPC patients did not distinguish patients in separate OS risk levels.

## Data Availability Statement 

The raw data supporting the conclusions of this article will be made available by the authors, without undue reservation.

## Ethics Statement

The studies involving human participants were reviewed and approved by Ethic committee of the University Hospital Frankfurt. Written informed consent for participation was not required for this study in accordance with the national legislation and the institutional requirements.

## Author Contributions

MW: Writing – Original Draft, Formal analysis, Methodology, Conceptualization. FP: Methodology, Visualization. BH: Formal analysis. MS: Formal analysis. CW: Conceptualization. TS: Writing – Review and Editing. HH: Writing – Review and Editing. SB: Writing – Review and Editing. MA: Writing – Review and Editing. AB: Writing – Review and Editing. PK: Writing – Review and Editing. FC: Writing – Review and Editing, Supervision. LK: Writing – Review and Editing, Validation. PM: Writing – Review and Editing, Supervision, Validation, Conceptualization. All authors contributed to the article and approved the submitted version.

## Conflict of Interest

The authors declare that the research was conducted in the absence of any commercial or financial relationships that could be construed as a potential conflict of interest.
